# Evolutionary origins of vocal mimicry in songbirds

**DOI:** 10.1002/evl3.62

**Published:** 2018-06-22

**Authors:** Maria Goller, Daizaburo Shizuka

**Affiliations:** ^1^ School of Biological Sciences University of Nebraska–Lincoln Lincoln Nebraska 68588‐0118

**Keywords:** vocal mimicry, vocal imitation, vocal learning, songbird, oscine

## Abstract

Vocal learning is an important behavior in oscines (songbirds). Some songbird species learn heterospecific sounds as well as conspecific vocalizations. The emergence of vocal mimicry is necessarily tied to the evolution of vocal learning, as mimicry requires the ability to acquire sounds through learning. As such, tracking the evolutionary origins of vocal mimicry may provide insights into the causes of variation in song learning programs among songbirds. We compiled a database of known vocal mimics that comprised 339 species from 43 families. We then traced the evolutionary history of vocal mimicry across the avian phylogeny using ancestral trait reconstruction on a dataset of oscine passerines for which vocalizations have been described. We found that the common ancestor to oscines was unlikely to mimic sounds, suggesting that song learning evolved with mechanisms to constrain learning to conspecific models. Mimicry then evolved repeatedly within the songbird clade, either through relaxation of constraints on conspecific learning or through selection for active vocal mimicry. Vocal mimicry is likely ancestral in only a handful of clades, and we detect many instances of independent origins of mimicry. Our analysis underscores the liability of vocal mimicry in songbirds, and highlights the evolutionary flexibility of song learning mechanisms.

**Impact statement**

Naturalists have long been fascinated by the ability of certain birds to imitate a wide range of sounds such as environmental noises and the vocalizations of other animals, including humans. This ability, often called vocal mimicry, is well known in some species (e.g., parrots, mockingbirds, lyrebirds). However, this ability is more widespread than often recognized, and provides clues to the diversity of song learning strategies within birds. Here, we built a database of 339 known flexible vocal mimics within the oscine passerines, known as ‘songbirds’. Using phylogenetic analysis on data from songbirds with described vocalizations, we traced the evolutionary origins of vocal mimicry. We show that vocal mimicry was probably not ancestral to songbirds, suggesting that the first songbirds likely sang only their own species’ song. This constraint to conspecific learning must have lifted in many clades through relaxation of learning constraints or selection for vocal mimicry for some beneficial function. Our study clearly demonstrates the evolutionary flexibility of vocal mimicry and song learning within songbirds.

Acoustic communication often plays a role in species recognition in animals (e.g., Emlen 1972; Claridge 1985; Hauber et al 2001; Seddon 2005; Percy et al. 2006). For many species, acoustic signals are genetically encoded; individuals can produce species‐specific sounds without the need for learning (Jang and Gerhardt 2006; Jarvis 2006; Janek and Slater 2000). In others, species‐specific vocalizations are learned by first perceiving acoustic information, and then practicing imitating these acquired sounds (Marler 1976). Such vocal learning is a behavior found in select mammals (humans, bats, cetaceans, and elephants; e.g., Jarvis 2006, Crockford et al. 2004; Prat et al. 2015) and birds (hummingbirds, parrots, and songbirds; e.g., Bolhuis and Gahr 2006). Within birds, this ability evolved independently multiple times (Tyack 2007; Jarvis 2007; Slater 1989).

Roughly half of the world's avian species are oscine passerines (songbirds) that learn their song. In many species, young birds listen to the songs of adults (gain an acoustic template), fine‐tune their imitation by comparing their practice songs to the acoustic template, and eventually produce a characteristic song of their own (much simplified; see Marler 1970a,b; Konishi 1965; Marler 1976; Soha 2017). However, there is striking variation in the oscine learning program (e.g., Nottebohm 1972; Soha 2017). Brenowitz and Beecher (2005) identified five dimensions of variation that cause complexity in song learning: timing of learning, number of songs learned, fidelity of imitation, type of exposure, and level of constraint to species‐specific models. The last dimension, level of constraint, ranges from species that learn only species‐specific song elements (highly constrained) to species that incorporate heterospecific and environmental sounds (unconstrained). In other words, a species can be discriminatory or permissive about what sounds it learns and incorporates into vocalizations.

Some highly permissive species, termed vocal mimics, readily learn not only species‐specific sounds but also sounds produced by other species or inanimate objects (Marshall 1950; Kaplan 2003; Kelley et al. 2008). There are many potential functions and definitions of vocal mimicry, which have been the focus of study (reviewed in Marshall 1950; Dobkin 1979; Baylis 1982; Hindmarsh 1986; Kelley et al. 2008; Dalziell et al. 2015; Jamie 2017; see also Dalziell and Welbergen 2016). However, little is known about the roots of mimetic ability in songbirds. Here, we focused on the evolutionary origins of the predilection to mimic heterospecific sounds, combined with the ability to imitate them (‘descriptive definition’ of vocal mimicry; Baylis 1982). Because very little is known about differences in the extent of mimicry across conspecifics, we assumed that mimicry is a species‐level trait to allow us to investigate the evolution of vocal mimicry.

The emergence of vocal mimicry is necessarily tied to the evolution of vocal learning, as mimicry requires the ability to acquire sounds through learning. Therefore, we assume that vocal mimicry could not have evolved before vocal learning. However, there are two broad scenarios in which vocal mimicry could have arisen relative to the emergence of vocal learning. First, if vocal learning evolved due to selection for increased song repertoire (e.g., Nottebohm and Liu 2010), mimicry could have evolved as a mechanism to acquire more song elements or as a by‐product of a broad acoustic template. The ability to mimic heterospecific sounds could then have been lost in lineages that evolved a narrow predisposition to learn only conspecific sounds. In this case, we would expect mimicry to align with the emergence of vocal learning and to be ancestral to all songbirds. Second, in an alternative scenario, vocal learning may have originally evolved with a strong bias toward acquisition of strictly species‐specific sounds. Over time, the perceptual window for sound acquisition could have become more permissive in some lineages, allowing vocal mimicry. The emergence of permissiveness in song learning could have arisen through relaxation of selection for narrow predispositions during song learning, or through strong selection for newly acquired functions (Dalziell et al. 2015).

To determine which of these two scenarios was more likely, we traced the evolutionary history of vocal mimicry across the avian phylogeny using ancestral trait reconstruction. First, we compiled a database of known mimetic species to better understand global patterns of mimicry. Second, we focused on the phylogenetic pattern of the emergence of mimicry. By tracing the history of vocal mimicry on the songbird phylogeny, we determined when the trait is likely to have emerged. Third, we used our phylogenetic approach to suggest the types of questions about mimicry that should be tackled in the future.

## Methods

### DEFINITION OF VOCAL MIMICRY

For the purposes of this study, we use a general definition of vocal mimicry, which encompasses imitation of all types of non‐conspecific sounds: other animals, anthropogenic (e.g., dog whistle, chainsaw), and environmental (e.g., water drip, leaves rustling) noises. We chose this definition to allow analysis of the evolution of the ability to learn and produce ‘mimicked’ sounds. While information on vocalizations is available for many species, determining the functions of mimicry requires careful experimental studies that have only been conducted on a few species. Here, we focused on the evolution of mimicry in species that utilize imitated sounds frequently and extensively, regardless of function.

### COMPILING THE DATABASE

Data were compiled from a variety of primary and secondary sources. A preliminary search was done on Google Scholar. We then expanded the search to various websites using search terms “mimic‐”, “imitate‐”, and “copy‐”. Field guides and handbooks, including all volumes of the Handbook of the Birds of the World, were browsed manually for mention of vocal imitation or copying behavior. Sources without peer‐review were verified whenever possible with an extensive search for corroborative scientific publications on each species on Google Scholar. If an account could not be verified, the species in question was not included in analysis. Scientific and common names were standardized using the IOC Bird List version 5.4 (Gill and Donsker 2015).

We compiled data on 557 oscine species to create our database of vocal mimicry (Supporting Information Table S1). We divided these species into three classifications corresponding to different extents of vocal imitation: “flexible”, “incidental”, and “unknown”. Under our classification scheme, “flexible mimics” were species that frequently imitate a wide variety of sounds, often having plastic repertoires and an extended period of song learning. In these species, mimetic ability is found in most individuals and is readily observed under natural conditions. In contrast, species that mimic on specific or rare occasions were classified as “incidental mimics”. This “incidental mimic” designation was also applied to mimicry in brood parasites that learn the calls or songs of their host species (17 species of *Vidua* finches; e.g., DaCosta and Sorenson 2014). Although brood parasites are flexible in which species they imitate (Langmore et al. 2008; Madden and Davies 2006), in this case, they have merely shifted their learning template from a conspecific to heterospecific tutor, and do not imitate a wider range of sounds (Kelley et al. 2008; DaCosta and Sorenson 2014). Other species considered “incidental mimics” were species of which individuals may have imitated a heterospecific in unnatural circumstances, such as when kept in captivity (e.g., bullfinches, zebra finches). Any accounts of a single individual imitating heterospecific song were also considered incidental mimicry, as this imitation most likely resulted from a learning mistake. The classification of “unknown mimic” was used for species reported to mimic but for which we found no details as to extent of imitation. In total, we classified 339 species as “flexible”, 120 as “incidental”, and 98 as “unknown” mimics.

We then classified oscine species for which we had found no accounts of mimicry as “non‐mimics”. However, to avoid scoring unstudied, potentially mimetic species as “non‐mimics”, we did not include species for which there were no data on vocalizations (based on accounts in the Handbook of the Birds of the World). Of the 5004 oscine species described, we found no information on the vocalizations of 594 species, and excluded these from all analyses. We therefore had a final dataset of 4410 oscine species (557 mimics and 3853 non‐mimics) for analysis.

To explore the robustness of our classification scheme, we treated each species as mimic or non‐mimic based on two alternative criteria (Supporting Information Figure S1). First, under our more conservative interpretation, we only considered flexible mimics as mimics, and treated incidental and unknown mimics as non‐mimics. We refer to this as the “flexible mimics” dataset. Second, we repeated our analyses using a more relaxed interpretation in which all three mimic classifications were treated as mimics. We refer to this as the “all mimics” dataset. We considered the first, more conservative, definition of mimicry (flexible mimics dataset) to be more reliable for evolutionary analysis of the use of mimicked sounds in vocal communication. However, using the second, more relaxed definition of mimicry (all mimics dataset) allowed us to test the robustness of our conclusions. In total, 339 flexible mimic species were considered mimics in the flexible mimic analysis, and an additional 218 species (for a total of 557 species) were scored as mimics in the analysis of all mimics.

### DESCRIPTIVE ANALYSIS

We calculated proportions of mimetic species within each avian family, as well as a global proportion, using the flexible and all mimics datasets. We were especially interested in patterns of mimicry within families, and determined whether mimics were clumped within, or dispersed throughout, a family. As we used published accounts of mimicking species, we expected under‐sampling of certain regions (Asia, Africa, and South America) compared to well‐studied ones (North America, Europe, and Australia). To investigate this further, we also calculated proportions of mimics based on geographic region. We used bird checklists from Avibase (excluding all accidental and introduced species) as the source for the total number of resident oscine species to allow comparison between regions (Lepage 2017).

### PHYLOGENETIC ANALYSIS

We conducted phylogenetic analyses using our compiled database to estimate the probability of mimicry being the ancestral state of songbirds. We first conducted analyses using alternate methods to assign species as vocal mimics. We downloaded the global phylogeny of birds accompanying Jetz et al. (2012). The tree source was Hackett All Species: a set of 1000 trees with 9993 operational taxonomic units (OTUs) each. As these trees included all avian species, we first pruned the trees by removing non‐oscine species. We then removed the 594 oscine species for which we found no song data in the Handbook of the Birds of the World. Our final phylogeny therefore included 4410 species. In the first analysis, we used the flexible mimics dataset and assigned a vocal mimic score of 1 to only the flexible mimic species, treating the incidental and unknown mimics as non‐mimics (score of 0). In our second analysis, we used our all mimics dataset in which flexible, incidental, and unknown mimics were all treated as vocal mimics (given a score of 1).

We compared the probability of mimicry being ancestral to oscines, and to each oscine family, based on these two analyses. The discrete character of mimicry was mapped onto the phylogeny as a basic binary value (present or absent). We then reconstructed the ancestral state for the basal ancestor of all species in the phylogeny, as well as the basal ancestor of each family. We reconstructed discrete ancestral states using the equal rates model, which estimates the marginal ancestral states based upon Bayesian likelihood. We also used stochastic character mapping to estimate the number of state changes across the phylogeny, also using the equal rates model. All analyses were done on the set of 1000 pruned trees using the R package *phytools* version 0.5‐38 (Revell 2012; Supporting Information).

We supplemented our phylogenetic analysis with two additional analyses to further explore the robustness of our results (see Supporting Information Methods for details). Both supplementary analyses incorporated only flexible mimics as mimics. One analysis restricted our dataset to the 817 oscines from regions with a long history of birdsong research (USA, Canada, Europe, Australia, and New Zealand). This regional analysis was meant to ensure that no species were mistakenly categorized as mimics or non‐mimics, as the vocalizations of every oscine from these regions have been described. The other analysis was on a dataset of 3550 avian species, including 65 non‐oscines and 3485 oscines. We included this analysis as a relatively unrestricted comparison that incorporated representatives from all avian orders.

## Results

### DESCRIPTIVE ANALYSIS

**Figure 1:**
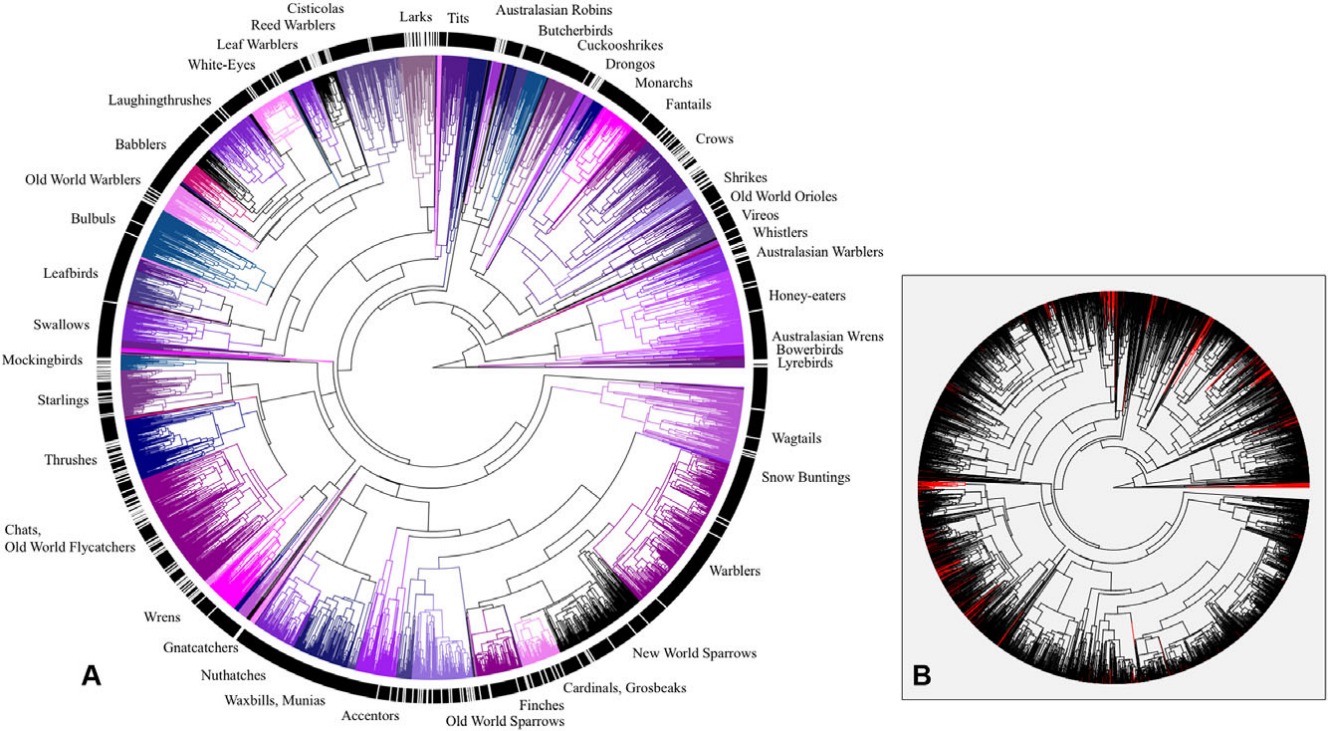
Phylogenetic tree of songbirds. (A) Presence of mimicry overlaid on phylogeny. Mimicry is represented by white marks in the dark ring. (B) Stochastic character mapping of estimated emergence and loss of mimetic ability. Mimicry is represented in red. Vocal mimicry evolved independently at least 237 times and was lost at least 52 times.

Of the roughly 5004 extant species and 115 families in the suborder Passeri (songbirds), 339 species (6.8%) from 43 families (37.4%) were classified as flexible mimics (Supporting Information Table S2). When considering only those 4410 oscine species for which we had vocalization data in the total, the percentage of mimetic oscine species becomes 8.9%. Songbird families vary greatly in mimetic ability and number of mimicking species (Fig. 1A and B). Using our dataset of flexible mimics, mimicry was rare (i.e., proportion of flexible mimics ≤ .10) in 95 of the 115 (82.6%) songbird families. In contrast, 16 families (13.9%) had a proportion of mimetic species between 0.1 and 0.5, while four songbird families (3.5%; Dicruridae, Nicatoridae, Ptilonorhynchidae, Menuridae) had a proportion higher than 0.5. In all cases, mimicry was spread across a family such that mimetic and non‐mimetic species were often each other's closest living relatives.

When using our more relaxed definition of mimicry (including incidental mimics and mimics with unknown extent of imitation), thirteen additional oscine families had at least one mimetic species, bringing the total to 56 families (48.7%). The number of families with proportions of mimicry between 0.1 and 0.5 jumped to 24 (20.9%), and the proportion of mimicking species in two additional families—Viduidae and Atrichornithidae—were above 0.5. The estimated global percentage of mimics based on this less restrictive analysis ranged from 11.0% (of the number of extant oscines) to 14.5% (of the pruned number of oscines included in analysis).

The proportion of oscine species that mimic varied regionally as well. In Europe, 31.6% of oscine species were flexible mimics, while other regions had 4–9% mimicking species (Table 2). These numbers nearly doubled when including all mimics, with percentages of mimics in North America, Australasia, and Africa rising to 10–15%, Europe rising to 60%, and other regions reaching 7%.

### PHYLOGENETIC ANALYSIS

Based on our conservative phylogenetic analysis constrained to flexible mimics, the most recent common ancestor of all songbirds most likely did not mimic (probability of presence = 0.129 ± 0.002; Table 1). When we use our more relaxed classification for mimics (all mimics dataset), our estimate of ancestral mimetic ability increases but remains unlikely (probability of presence = 0.220 ± 0.002). Our results remain qualitatively similar in two other versions of the phylogenetic analysis. The probability that the common ancestor of songbirds was mimetic is low when we constrain our analysis to geographic regions in which birdsong has been particularly well‐studied (probability of presence = 0.167 ± 0.39), and when we include all songbirds (probability of presence = 0.185 ± 0.003).

**Table 1:** Probability of mimicry as the ancestral trait in oscines based on classification scheme

Dataset	Classifications scored as mimic	Number of mimics	Total number of species	Probability of ancestral mimicry
Flexible mimics	Flexible	339	4410	0.129 ± 0.002
All mimics	Flexible, incidental, unknown	557	4410	0.220 ± 0.002
Regional	Flexible	339	817	0.167 ± 0.39
Unrestricted	Flexible	339	3550	0.185 ± 0.003

When using our flexible mimic dataset, we estimate that vocal mimicry evolved independently at least 237 times across oscine taxa (Fig. 1B) and was lost 52 times on average. These values become 401 and 100, respectively, when using the all mimics database. The ancestors of three songbird families—Mimidae, Dicruridae, Menuridae—were likely vocal mimics (probability ≈ 0.75 or greater; Table 3). It is unclear whether mimicry was ancestral to Atrichornithidae, Ptilonorhynchidae, and Nicatoridae (probability ≈ 0.5). All other oscine families did not have ancestors that actively mimicked (*P* < 0.05, Supporting Information Table S2). Results were similar in the analysis using all mimics, although under this relaxed definition mimicry was likely ancestral to Atrichornithidae and Ptilonorhynchidae as well.

## Discussion

With high likelihood, mimetic ability was not ancestral to songbirds. Instead, vocal mimicry evolved numerous times within oscines and is currently widespread among extant species. One‐third of songbird families contain at least one mimicking species, and there are a few families in which most species are mimetic. Our phylogenetic analysis indicates that mimicry may have emerged at the base of some families, multiple times within other families, or never emerged in still other families. Mimicry appears to have been ancestral to two families (Dicruridae and Menuridae), with probabilities of presence greater than 0.75. These families include species in which functions of mimicry have been well studied. For example, greater racket‐tailed drongos, *Dicrurus paradiseus*, may use mimicked alarm calls of heterospecific flock members to demonstrate aggressive intent or to steal food (e.g., Satischandra et al. 2010). In superb lyrebirds, *Menura novaehollandiae*, mimetic accuracy may be used by females to choose between males (Dalziell and Magrath 2012; Coleman et al. 2007). However, although mimicry clearly serves a function in certain clades, it is unclear whether such functions alone can account for the widespread emergence of vocal mimicry across oscines.

Our global estimate of flexible mimics ranges from approximately 7 to 9%. However, there is strong geographic variation in the prevalence of vocal mimicry. For example, while 31.6% of European oscines were categorized as flexible mimics, all other regions only had an average of 6% flexible mimetic species. Furthermore, the pattern changes within regions. While the percentage of flexible mimics in Australia and New Zealand was 14.9% (see also Chisholm 1932; Marshall 1950), this percentage became 7.5% for all Australasian islands. Similarly, the percentage of North American mimics increases from 7.1 to 14.2% when excluding Mexican species. Part of this difference may be explained by a dearth of publications on the vocalizations of understudied avifauna in certain regions. However, although we expect that our knowledge of mimics is more complete for certain regions, this cannot fully explain the difference. For example, although our number of mimicking North American species is relatively small, we believe this number to be accurate, as North American oscines have been studied extensively. Although we have no reason a priori for expecting Europe to have a large percentage of mimics, further study is needed to understand this pattern.

There has long been debate over the definition of vocal mimicry (reviewed in Dalziell et al. 2015). Here, we focused on evidence for flexible use of imitated sounds (e.g., heterospecific, anthropogenic, environmental) based on existing literature. However, we acknowledge that there is uncertainty associated with how to categorize and define mimetic species. To account for this uncertainty, we also assessed the prevalence of species with records of incidental mimicry (e.g., evidence of heterospecific copying in captivity or other limited contexts) as well as species for which accounts of vocal mimicry did not specify the extent of flexibility. Many of these species are unlikely to be considered vocal mimics under any definition, but including incidental mimics provided a maximum estimate of vocally mimicking species. When including these putative mimics (‘all mimics’ in Tables 1 and 2), we estimate that approximately 11–15% of oscine species imitate non‐conspecific sounds.

**Table 2:** Proportion of mimics on each continent

Region	Total oscine species	Number of flexible mimics (proportion of total)	Number of all mimics (proportion of total)
North America	762	54 (0.071)	85 (**0.112**)
Europe	215	68 (**0.316**)	129 (**0.600**)
Australasia	778	58 (0.075)	87 (**0.112**)
Asia	2004	90 (0.045)	137 (0.068)
Africa	1442	132 (0.092)	222 (**0.154**)
South America	825	32 (0.039)	61 (0.074)
Central America	531	26 (0.049)	41 (0.077)

**Table 3:** Probabilities of the presence of vocal mimicry in the ancestor of select songbird families

Oscine family	Species (in analysis)	Species (total)	Proportion of species (in analysis)	Mimic species (flexible)	Proportion flexible mimic (of total)	Mimic species (all)	Proportion all mimic (total)	Probability ancestor was mimic (flexible)	Probability ancestor was mimic (all)
Atrichornithidae	2	2	1	1	0.5	2	1	0.574 ± 0.005	0.92 ± 0.003
Dicruridae	21	25	0.84	13	0.52	17	0.68	0.854 ± 0.006	0.831 ± 0.005
Menuridae	2	2	1	2	1	2	1	0.949 ± 0.002	0.926 ± 0.003
Mimidae	34	34	1	16	0.47	17	0.5	0.742 ± 0.004	0.635 ± 0.003
Nicatoridae	3	3	1	2	0.67	2	0.67	0.417 ± 0.007	0.48 ± 0.006
Ptilonorhynchidae	17	20	0.85	14	0.7	17	0.85	0.576 ± 0.008	0.748 ± 0.005

However, we (and most others to date) have treated vocal mimicry as a species‐level trait. Our literature review revealed little information on within‐species variation in vocal mimicry. Given the evolutionary lability of this trait, it is likely that the extent of vocal mimicry varies within some species. Although we cannot account for any within‐species variation in our analysis, understanding the variation in extent and function of vocal mimicry within species would yield further understanding of the evolutionary origins of vocal mimicry.

Our phylogenetic analysis indicates that vocal mimicry was likely not ancestral to oscines. As song learning probably evolved at some point early in the evolution of oscines (Nottebohm 1972; Nottebohm and Liu 2010), this implies that mimetic ability did not evolve concurrently with the origin of song learning. Instead, our analysis supports the hypothesis that the ancestral songbird had a restricted song template that excluded non‐species‐specific sounds. Vocal flexibility may have been limited by constraints on template acquisition facilitating the learning of only conspecific sounds, and/or by restrictive sound production mechanisms. These restrictions and constraints on vocal mimicry would have lessened repeatedly and independently within the songbird clade.

Given our results, the question becomes why and how restrictions on sensory recognition (song template) and/or sound production (syringeal function) became relaxed in some lineages. Here, we propose two hypotheses. In the first hypothetical scenario, species‐specificity in both song recognition and production slowly relaxed over time in the absence of selection. At some point after song learning evolved, mimicry became possible and the imitation of heterospecific sounds became commonplace in many species, eventually gaining functional significance. Alternatively, permissiveness in imitation may have undergone repeated positive selection after the evolution of song learning. A proposed mechanism driving the evolution of vocal learning is mate choice based on song complexity or novelty (Nottebohm 1972; Jarvis 2004) as females of many species appear to prefer males singing more complex repertoires (e.g., canaries, Draganoiu et al 2002; starlings, Mountjoy and Lemon 1996; Gentner and Hulse 2000; chaffinches, Leitao et al 2005), and learning enhances complexity (Nottebohm 1972; Jarvis 2006). Similarly, Laiolo et al. (2011) suggest that mimicry increases song complexity and serves as an honest signal. As such, selection for vocal repertoire complexity or plasticity in vocal performance may have led to a less restricted song learning template. As imitation became increasingly plastic, vocal learning would have broadened to include mimicry. Of the mimicking species used in our analysis, more than 90% are likely using mimicry solely in song. It therefore seems likely that sexual selection played a role in the emergence of mimicry.

Vocal mimicry requires both a broadly permissive song template (or lack of template) and the morphological ability to produce imitations. Yet, the imitation of heterospecific sounds has evolved frequently, suggesting that relatively simple mechanisms may separate non‐mimetic and mimetic species. This also suggests that some species may have the capability to mimic but only express the trait under certain circumstances. Social context may be one important factor driving incidental mimicry of heterospecifics, at least in captive conditions (as with starlings, West and King 1990; bullfinches, Nicolai 1959; budgerigars, Gramza 1970). In our database, the incidental mimic classification includes species that imitated when kept without conspecific social partners, but are not known to imitate in the wild (e.g., zebra finch and Oregon junco, Bertram 1970; house sparrow, Conradi 1905). Similarly, another factor influencing incidental mimicry could be similarity between vocalizations of conspecifics and heterospecifics, such that learning of heterospecific song can occur by accident (Kelley et al. 2008; e.g., short‐toed and Eurasian treecreepers, Garamszegi et al. 2007; Thielcke 1962, 1972). These examples underscore the diversity of forms vocal mimicry can take, suggesting that mimicry is not a discrete trait, but rather a continuum within the spectrum of vocal flexibility. Therefore, one further way to explore the evolution of vocal mimicry may be to study transitions between incidental and flexible mimics. Similarly, studying pairs of sister species, one mimetic and one non‐mimetic, may improve our understanding of vocal mimicry. More generally, we suggest that phylogenetic and comparative approaches to vocal flexibility can continue to provide insights into the evolution of avian song learning programs.

## DATA ACCESSIBILITY

The full database, methods, and supplemental R script can be found in Supporting Information Materials.

Associate Editor: A. Goswami

## Supporting information


**Appendix I**.

**Figure S1**

**Table S1**

**Table S2**

Supporting information
