# Supplementary material for: Evolutionary origins of vocal mimicry in songbirds
Source: Evol Lett. 2018 Jun 22;2(4):417–26. doi: 10.1002/evl3.62 (PMC6121844; doi:10.1002/evl3.62)
Supplement: Supplementary file 1 — Appendix I. [file EVL3-2-417-s001.docx]

**Appendix I.** Full list of recordings used from xenocanto and Macaulay library.

Xenocanto:

Woxvold, I. 2008. Rusty Mouse-warbler (*Crateroscelis murina*). XC87564. http://www.xeno-canto.org/87564.

Lachmann, L. 2017. Melodious warbler (*Hippolais polyglotta*). XC375259. http://www.xeno-canto.org/375259.

Calvet, J. 2013. Melodious warbler (*Hippolais polyglotta*). XC134271. http://www.xeno-canto.org/134271.

Dragonetti, M. 2009. Melodious warbler (*Hippolais polyglotta*). XC44642, XC44641. http://www.xeno-canto.org/44642.

Krajenbrink, H. 2015. Melodious warbler (*Hippolais polyglotta*). XC252579, XC252578. http://www.xeno-canto.org/252579.

Anderson, M. 2017. Redthroat (*Pyrrholaemus brunneus*). XC383695. ttp://www.xeno-canto.org/383695.

Carter, M. 2014. Redthroat (*Pyrrholaemus brunneus*). XC206881. http://www.xeno-canto.org/206881.

Anderson, M. 2014. Redthroat (*Pyrrholaemus brunneus*). XC200388. http://www.xeno-canto.org/200388.

McGeehan, A. 2015. Eurasian skylark (*Alauda arvensis*). XC241900, XC241888. http://www.xeno-canto.org/241900.

Joshi, V. 2011. Oriental skylark (*Alauda gulgula*). XC86702. http://www.xeno-canto.org/86702.

Peacock, F. 2017. Large-billed lark (*Galerida magnirostris*). XC377628. http://www.xeno-canto.org/377628.

Collett, A. 2011. Large-billed lark (*Galerida magnirostris*). XC78969. http://www.xeno-canto.org/78969.

Slaymaker, M. 2013. Asian short-toed lark (*Alauda cheleensis*). XC143025, XC143023. http://www.xeno-canto.org/143025.

Graves, T.G. 2014. Steller's jay (*Cyanocitta stelleri*). XC170836. http://www.xeno-canto.org/170836.

Cruickshank, I. 2011. Steller's jay (*Cyanocitta stelleri*). XC161655. http://www.xeno-canto.org/161655.

Lambert, F. 2012. Tibetan lark (*Melanocorypha maxima*). XC110990, XC110991, XC110992. http://www.xeno-canto.org/110990.

Dharwadkar, O. 2013. Golden-fronted leafbird (*Chloropsis aurifrons*). XC190755. http://www.xeno-canto.org/190755.

Elman, U. 2017. Eurasian jay (*Garrulus glandarius*). XC366336, XC366337. http://www.xeno-canto.org/366336.

Deoniziak, K. 2016. Eurasian jay (*Garrulus glandarius*). XC312912. http://www.xeno-canto.org/312912.

Matusiak, J. 2015. Eurasian jay (*Garrulus glandarius*). XC287827. http://www.xeno-canto.org/287827.

Matusiak, J. 2014. Eurasian jay (*Garrulus glandarius*). XC169039. http://www.xeno-canto.org/169039.

Matusiak, J. 2013. Eurasian jay (*Garrulus glandarius*). XC121679. http://www.xeno-canto.org/121679.

Aberg, P. 2011. Eurasian jay (*Garrulus glandarius*). XC84021, XC84020. http://www.xeno-canto.org/84021.

Fisher, S. 2011. Eurasian jay (*Garrulus glandarius*). XC74409. http://www.xeno-canto.org/74409.

Matusiak, J. 2010. Eurasian jay (*Garrulus glandarius*). XC46762, XC45004. http://www.xeno-canto.org/46762.

Livon. 2017. Eurasian jay (*Garrulus glandarius*). XC394409. http://www.xeno-canto.org/394409.

Torimi, A. 2017. Eurasian jay (*Garrulus glandarius*). XC362124. http://www.xeno-canto.org/362124.

Elman, U. 2017. Eurasian jay (*Garrulus glandarius*). XC361068, XC361067, XC361066, XC361065. http://www.xeno-canto.org/361068.

Holzapfel, F. 2017. Eurasian jay (*Garrulus glandarius*). XC356862. http://www.xeno-canto.org/356862.

Torimi, A. 2016. Eurasian jay (*Garrulus glandarius*). XC310769, XC309519, XC309518, XC309517. http://www.xeno-canto.org/310769.

Sonnenburg. 2016. Eurasian jay (*Garrulus glandarius*). XC308733. http://www.xeno-canto.org/308733.

Elman, U. 2016. Eurasian jay (*Garrulus glandarius*). XC308727, XC308726. http://www.xeno-canto.org/308727.

Bouglouan, N. 2015. Eurasian jay (*Garrulus glandarius*). XC234079. http://www.xeno-canto.org/234079.

Torimi, A. 2015. Eurasian jay (*Garrulus glandarius*). XC233686, XC217776. http://www.xeno-canto.org/233686.

Krajenbrink, H. 2014. Eurasian jay (*Garrulus glandarius*). XC215618. http://www.xeno-canto.org/215618.

van der Meer, H. 2014. Eurasian jay (*Garrulus glandarius*). XC178457. http://www.xeno-canto.org/178457.

van der Meer, H. 2013. Eurasian jay (*Garrulus glandarius*). XC131112, XC129155. http://www.xeno-canto.org/131112.

Aberg, P. 2012. Eurasian jay (*Garrulus glandarius*). XC107269. http://www.xeno-canto.org/107269.

Szczypinski, P. 2017. Eurasian jay (*Garrulus glandarius*). XC397350. http://www.xeno-canto.org/397350.

Fisher, S. 2017. Eurasian jay (*Garrulus glandarius*). XC390269. http://www.xeno-canto.org/390269.

Szczypinski, P. 2017. Eurasian jay (*Garrulus glandarius*). XC357499. http://www.xeno-canto.org/357499.

Dvorak, M. 2015. Eurasian jay (*Garrulus glandarius*). XC215709. http://www.xeno-canto.org/215709.

Torimi, A. 2014. Eurasian jay (*Garrulus glandarius*). XC201559, XC194039. http://www.xeno-canto.org/201559.

Jongsma, J. 2015. Grey jay (*Perisoreus canadensis*). XC254435. http://www.xeno-canto.org/254435.

Hoyer, R. 2014. Grey jay (*Perisoreus canadensis*). XC187488. http://www.xeno-canto.org/187488.

Matusiak, J. 2012. Eurasian magpie (*Pica pica*). XC113580. http://www.xeno-canto.org/113580.

Chie-Jen, K. 2014. Bronzed drongo (*Dicrurus aeneus*). XC207258. http://www.xeno-canto.org/207258.

Chie-Jen, K. 2011. Bronzed drongo (*Dicrurus aeneus*). XC76308. http://www.xeno-canto.org/76308.

Edwards, D. 2008. Greater racket-tailed drongo (*Dicrurus paradiseus*). XC25969. http://www.xeno-canto.org/25969.

DeFonso, E. 2016. Greater racket-tailed drongo (*Dicrurus paradiseus*). XC372568. http://www.xeno-canto.org/372568.

Dharwadkar, O. 2014. Greater racket-tailed drongo (*Dicrurus paradiseus*). XC190842. http://www.xeno-canto.org/190842.

Patel, Y. 2016. Greater racket-tailed drongo (*Dicrurus paradiseus*). XC317402. http://www.xeno-canto.org/317402.

Puliyeri, V. 2015. Greater racket-tailed drongo (*Dicrurus paradiseus*). XC296428. http://www.xeno-canto.org/296428.

Bradley, D. 2007. Greater racket-tailed drongo (*Dicrurus paradiseus*). XC24074. http://www.xeno-canto.org/24074.

Roy, S. Lesser racket-tailed drongo (*Dicrurus remifer*). XC90391. http://www.xeno-canto.org/90391.

Spencer, A. 2011. White-rimmed brush finch (*Atlapetes leucopis*). XC87019. http://www.xeno-canto.org/87019.

Webster, R. 2008. Green-tailed towhee (*Pipilo chlorurus*). XC112017. http://www.xeno-canto.org/112017.

Rigby, J.R. 2015. Eastern towhee (*Pipilo erythrophthalmus*). XC247525. http://www.xeno-canto.org/247525.

Gregory, P. 2013. Spangled drongo (*Dicrurus bracteatus*). XC121029. http://www.xeno-canto.org/121029.

Litsgard, M. 2017. Whinchat (*Saxicola rubetra*). XC371512. http://www.xeno-canto.org/371512.

Elman, U. 2015. Whinchat (*Saxicola rubetra*). XC288932. http://www.xeno-canto.org/288932.

Szczypinski, P. 2015. Whinchat (*Saxicola rubetra*). XC239443. http://www.xeno-canto.org/239443.

Matusiak, J. 2012. Whinchat (*Saxicola rubetra*). XC100991. http://www.xeno-canto.org/100991.

Fisher, S. 2013. Whinchat (*Saxicola rubetra*). XC139594. http://www.xeno-canto.org/139594.

Goujon, G. 2011. Whinchat (*Saxicola rubetra*). XC94907. http://www.xeno-canto.org/94907.

Austin, T. 2016. Yellow-breasted chat (*Icteria virens auricollis*). XC313669. http://www.xeno-canto.org/313669.

Olmstead, S. 2014. Yellow-throated euphonia (*Euphonia hirundinacea*). XC209852. http://www.xeno-canto.org/209852.

Brooks, T. 2011. Thick-billed euphonia (*Euphonia laniirostris*). XC108063. http://www.xeno-canto.org/108063.

Spencer, A. 2010. Thick-billed euphonia (*Euphonia laniirostris*). XC45661. http://www.xeno-canto.org/45661.

de Godoy, F.I. 2011. Violaceous euphonia (*Euphonia violacea*). XC323961. http://www.xeno-canto.org/323961.

DeFonso, E. 2014. Violaceous euphonia (*Euphonia violacea*). XC242057, XC242051, XC241797, XC207276. http://www.xeno-canto.org/242057.

Athanas, N. 2006. Violaceous euphonia (*Euphonia violacea*). XC11552. http://www.xeno-canto.org/11552.

Jones, D. 1993. Violaceous euphonia (*Euphonia violacea*). XC1341. http://www.xeno-canto.org/1341.

Souza, R. 2015. Violaceous euphonia (*Euphonia violacea*). XC265096. http://www.xeno-canto.org/265096.

Brito, G.R.R. 2005. Violaceous euphonia (*Euphonia violacea*). XC20329. http://www.xeno-canto.org/20329.

Davis, T. 2013. White-eyed vireo (*Vireo griseus*). XC215875. http://www.xeno-canto.org/215875.

Cruickshank, I. 2010. Purple finch (*Haemorhous purpureus*). XC161662. http://www.xeno-canto.org/161662.

Szczypinski, P. 2014. Red crossbill (*Loxia curvirostra*). XC167651. http://www.xeno-canto.org/167651.

Grahn, J. 2013. Red crossbill (*Loxia curvirostra*). XC161609. http://www.xeno-canto.org/161609.

Lane, D. 2012. Greenish yellow finch (*Sicalis olivascens*). XC105093. http://www.xeno-canto.org/105093.

Harter, L. 2013. Lawrence's goldfinch (*Spinus lawrencei*). XC134010. http://www.xeno-canto.org/134010.

Lane, D. 2015. Hooded siskin (*Spinus magellanica*). XC272329, XC272330. http://www.xeno-canto.org/272329.

Pluym, D.V. 2015. Black-headed siskin (*Spinus notata*). XC302539. http://www.xeno-canto.org/302539.

Lane, D. 1989. Black-headed siskin (*Spinus notata*). XC28791. http://www.xeno-canto.org/28791.

Wilson, T. 2011. Pine siskin (*Spinus pinus*). XC71592. http://www.xeno-canto.org/71592.

Harter, L. 2013. Lesser goldfinch (*Spinus psaltria*). XC144120, XC144129. http://www.xeno-canto.org/144120.

Grosselet, M. 2015. Lesser goldfinch (*Spinus psaltria*). XC254541. http://www.xeno-canto.org/254541.

Lane, D. 2013. Lesser goldfinch (*Spinus psaltria*). XC146611. http://www.xeno-canto.org/146611.

Grosselet, M. 2010. Lesser goldfinch (*Spinus psaltria*). XC62693, XC55398. http://www.xeno-canto.org/62693.

Spencer, A. 2007. Lesser goldfinch (*Spinus psaltria*). XC14548. http://www.xeno-canto.org/14548.

Harter, L. 2013. Lesser goldfinch (*Spinus psaltria*). XC121125. http://www.xeno-canto.org/121125.

DeFonso, E. 2010. Lesser goldfinch (*Spinus psaltria*). XC102954. http://www.xeno-canto.org/102954.

Grosselet, M. 2011. Lesser goldfinch (*Spinus psaltria*). XC70444. http://www.xeno-canto.org/70444.

Szczypinski, P. 2014. Eurasian siskin (*Spinus spinus*). XC336189, XC169740. http://www.xeno-canto.org/336189.

Davis, T. 2015. Red-winged blackbird (*Agelaius phoeniceus*). XC254764. http://www.xeno-canto.org/254764.

Moore, J.V. 1995. Yellow-rumped cacique (*Cacicus cela*). XC259952. http://www.xeno-canto.org/259952.

Gomez, O.H.M. 2015. Yellow-rumped cacique (*Cacicus cela*). XC245378, XC245379, XC245380, XC245490, XC245489. http://www.xeno-canto.org/245378.

Ingremeau, P. 2014. Yellow-rumped cacique (*Cacicus cela*). XC213676. http://www.xeno-canto.org/213676.

Lane, D. 2004. Yellow-rumped cacique (*Cacicus cela*). XC86563. http://www.xeno-canto.org/86563.

Lane, D. 2008. Yellow-rumped cacique (*Cacicus cela*). XC66024, XC65977. http://www.xeno-canto.org/66024.

Ingremeau, P. Yellow-rumped cacique (*Cacicus cela*). XC27272. http://www.xeno-canto.org/27272.

DeFonso, E. 2011. Yellow-rumped cacique (*Cacicus cela*). XC122293. http://www.xeno-canto.org/122293.

Lane, D. 2013. Epaulet oriole (*Icterus cayanensis*). XC158341. http://www.xeno-canto.org/158341.

Torimi, A. 2017. Bull-headed shrike (*Lanius bucephalus*). XC358000. http://www.xeno-canto.org/358000.

Torimi, A. 2016. Bull-headed shrike (*Lanius bucephalus*). XC343003. http://www.xeno-canto.org/343003.

Torimi, A. 2015. Bull-headed shrike (*Lanius bucephalus*). XC225376. http://www.xeno-canto.org/225376.

Ko, C-J. 2010. Long-tailed shrike (*Lanius schach*). XC62705. http://www.xeno-canto.org/62705.

Trainor, C. 2010. Long-tailed shrike (*Lanius schach*). XC103149. http://www.xeno-canto.org/103149.

McCafferty, A. 2009. Woodchat shrike (*Lanius senator*). XC37569. http://www.xeno-canto.org/37569.

Lin, S. 2014. Black-throated laughingthrush (*Garrulax chinensis*). XC178247. http://www.xeno-canto.org/178247.

Grosselet, M. 2015. Blue mockingbird (*Melanotis caerulescens*). XC249305, XC288235, XC249301. http://www.xeno-canto.org/249305.

Lane, D. 2016. Blue mockingbird (*Melanotis caerulescens*). XC302854. http://www.xeno-canto.org/302854.

van Dort, J. 2015. Blue-and-white mockingbird (*Melanotis hypoleucus*). XC237359. http://www.xeno-canto.org/237359.

O'Donnell, P. 2013. Tropical mockingbird (*Mimus gilvus*). XC169753. http://www.xeno-canto.org/169753.

Lane, D. 2001. Chalk-browed mockingbird (*Mimus saturninus*). XC145450. http://www.xeno-canto.org/145450.

Lane, D. 2014. Curve-billed thrasher (*Toxostoma curvirostre*). XC208826. http://www.xeno-canto.org/208826.

Pluym, D.V. 2014. Curve-billed thrasher (*Toxostoma curvirostre*). XC170661. http://www.xeno-canto.org/170661.

Lane, D. 2016. Ocellated thrasher (*Toxostoma ocellatum*). XC302852. http://www.xeno-canto.org/302852.

Lane, D. 2009. Ocellated thrasher (*Toxostoma ocellatum*). XC67055. http://www.xeno-canto.org/67055.

Marvin, P. 2015. California thrasher (*Toxostoma redivivum*). XC283685. http://www.xeno-canto.org/283685.

Trainor, C. 2009. Lesser shortwing (*Brachypteryx leucophris*). XC103157. http://www.xeno-canto.org/103157.

Lambert, F. 2012. Lesser shortwing (*Brachypteryx leucophris*). XC100851. http://www.xeno-canto.org/100851.

Allen, D. 1994. Black shama (*Copsychus cebuensis*). XC79185. http://www.xeno-canto.org/79185.

Nefdt, R. 2012. Blue-shouldered robin-chat (*Cossypha cyanocampter*). XC98710, XC98701. http://www.xeno-canto.org/98710.

Kilusu, I. 2017. Rüppell's robin-chat (*Cossypha semirufa*). XC391599. http://www.xeno-canto.org/391599.

Matheve, H. 2012. Rüppell's robin-chat (*Cossypha semirufa*). XC154735. http://www.xeno-canto.org/154735.

Nefdt, R. 2012. Rüppell's robin-chat (*Cossypha semirufa*). XC98799. http://www.xeno-canto.org/98799.

Bradley, J. 2015. Rüppell's robin-chat (*Cossypha semirufa*). XC289776, XC289775. http://www.xeno-canto.org/289776.

Carter, S. 2013. Rüppell's robin-chat (*Cossypha semirufa*). XC236414, XC236413, XC236412. http://www.xeno-canto.org/236414.

Bradley, J. 2014. Rüppell's robin-chat (*Cossypha semirufa*). XC205517. http://www.xeno-canto.org/205517.

Nefdt, R. 2012. Rüppell's robin-chat (*Cossypha semirufa*). XC98797, XC98796. http://www.xeno-canto.org/98797.

Torimi, A. 2016. Blue-and-white flycatcher (*Cyanoptila cyanomelana*). XC313294, XC311867, XC311618, XC318690, XC311613. http://www.xeno-canto.org/313294.

Torimi, A. 2014. Blue-and-white flycatcher (*Cyanoptila cyanomelana*). XC191097, XC191086, XC194503, XC192103, XC190251, XC190154. http://www.xeno-canto.org/191097.

Torimi, A. 2017. Blue-and-white flycatcher (*Cyanoptila cyanomelana*). XC364133. http://www.xeno-canto.org/364133.

Torimi, A. 2015. Blue-and-white flycatcher (*Cyanoptila cyanomelana*). XC236741. http://www.xeno-canto.org/236741.

Litsgard, M. 2017. Common redstart (*Phoenicurus phoenicurus*). XC371508. http://www.xeno-canto.org/371508.

Elman, U. 2016. Common redstart (*Phoenicurus phoenicurus*). XC336545. http://www.xeno-canto.org/336545.

Elman, U. 2015. Common redstart (*Phoenicurus phoenicurus*). XC288947. http://www.xeno-canto.org/288947.

Litsgard, M. 1981. Common redstart (*Phoenicurus phoenicurus*). XC211837. http://www.xeno-canto.org/211837.

Szymanski, P. 2017. Common redstart (*Phoenicurus phoenicurus*). XC367523. http://www.xeno-canto.org/367523.

Litsgard, M. 1978. Common redstart (*Phoenicurus phoenicurus*). XC211834. http://www.xeno-canto.org/211834.

Szymanski, P. 2017. Common redstart (*Phoenicurus phoenicurus*). XC367524.http://www.xeno-canto.org/367524.

Fisher, S. 2007. European stonechat (*Saxicola rubicola*). XC25601. http://www.xeno-canto.org/25601.

Trainor, C. 2005. Green figbird (*Sphecotheres viridis*). XC32600. http://www.xeno-canto.org/32600.

Holzapfel, F. 2017. Great tit (*Parus major*). XC367168. http://www.xeno-canto.org/367168.

Matusiak, J. 2015. Great tit (*Parus major*). XC233398. http://www.xeno-canto.org/233398.

Paal, U. 2015. Great tit (*Parus major*). XC215446. http://www.xeno-canto.org/215446.

Matusiak, J. 2013. Great tit (*Parus major*). XC129215, XC129195, XC127374, XC126692, XC125448, XC125444, XC123531. http://www.xeno-canto.org/129215.

Harter, L. 2014. MacGillivray's warbler (*Geothlypis tolmiei*). XC187113. http://www.xeno-canto.org/187113.

Hoyer, R.C. 2012. White-lored gnatcatcher (*Polioptila albiloris*). XC97463. http://www.xeno-canto.org/97463.

Jongsma, J. 2014. Blue-grey gnatcatcher (*Polioptila caerulea*). XC178125. http://www.xeno-canto.org/178125.

Driver, P. 2013. Blue-grey gnatcatcher (*Polioptila caerulea*). XC174835, XC174833. http://www.xeno-canto.org/174835.

Lane, D. 1994. Blue-grey gnatcatcher (*Polioptila caerulea*). XC135063. http://www.xeno-canto.org/135063.

Elias, E.A. 2014. Black-capped gnatcatcher (*Polioptila nigriceps*). XC186035. http://www.xeno-canto.org/186035.

Hoyer, R.C. 2011. Black-capped gnatcatcher (*Polioptila nigriceps*). XC76315, XC76314, XC76313. http://www.xeno-canto.org/76315.

DeFonso, E. 2014. Phainopepla (*Phainopepla nitens*). XC173177, XC173175. http://www.xeno-canto.org/173177.

de Molina, J.M. 2009. Spotless starling (*Sturnus unicolor*). XC32155, XC32153, XC32154. http://www.xeno-canto.org/32155.

Litsgard, M. 2017. Eurasian blackcap (*Sylvia atricapilla*). XC375220. http://www.xeno-canto.org/375220.

Ryberg, E.A. 2017. Eurasian blackcap (*Sylvia atricapilla*). XC367355. http://www.xeno-canto.org/367355.

Hamann, A. 2017. Eurasian blackcap (*Sylvia atricapilla*). XC366482. http://www.xeno-canto.org/366482.

Rochefort, J. 2015. Eurasian blackcap (*Sylvia atricapilla*). XC314032. http://www.xeno-canto.org/314032.

Litsgard, M. 2015. Eurasian blackcap (*Sylvia atricapilla*). XC288128. http://www.xeno-canto.org/288128.

Pron, M. 2014. Eurasian blackcap (*Sylvia atricapilla*). XC186628. http://www.xeno-canto.org/186628.

Szczypinski, P. 2014. Eurasian blackcap (*Sylvia atricapilla*). XC186197. http://www.xeno-canto.org/186197.

van der Meer, H. 2014. Eurasian blackcap (*Sylvia atricapilla*). XC179508. http://www.xeno-canto.org/179508.

Krabbe, N. 2011. Eurasian blackcap (*Sylvia atricapilla*). XC77468. http://www.xeno-canto.org/77468.

Dragonetti, M. 2008. Eurasian blackcap (*Sylvia atricapilla*). XC44645. http://www.xeno-canto.org/44645.

Mroczko, C. 2017. Eurasian blackcap (*Sylvia atricapilla*). XC385521. http://www.xeno-canto.org/385521.

Hansen, L.A. 2017. Eurasian blackcap (*Sylvia atricapilla*). XC384258, XC384229, XC378312. http://www.xeno-canto.org/384258.

Grosselet, M. 2017. Eurasian blackcap (*Sylvia atricapilla*). XC374621. http://www.xeno-canto.org/374621.

Hamann, A. 2017. Eurasian blackcap (*Sylvia atricapilla*). XC369934, XC366483. http://www.xeno-canto.org/369934.

Hansen, L.A. 2010. Eurasian blackcap (*Sylvia atricapilla*). XC365631, XC325188. http://www.xeno-canto.org/365631.

Malengreau, A. 2014. Eurasian blackcap (*Sylvia atricapilla*). XC302827. http://www.xeno-canto.org/302827.

van Bruggen, J. 2015. Eurasian blackcap (*Sylvia atricapilla*). XC292854. http://www.xeno-canto.org/292854.

Matusiak, J. 2014. Eurasian blackcap (*Sylvia atricapilla*). XC184491. http://www.xeno-canto.org/184491.

Szczypinski, P. 2014. Eurasian blackcap (*Sylvia atricapilla*). XC182602, XC182599, XC182523, XC182522. http://www.xeno-canto.org/182602.

Peron, M. 2013. Eurasian blackcap (*Sylvia atricapilla*). XC172874. http://www.xeno-canto.org/172874.

Grosselet, M. 2013. Eurasian blackcap (*Sylvia atricapilla*). XC134355. http://www.xeno-canto.org/134355.

Goujon, G. 2011. Eurasian blackcap (*Sylvia atricapilla*). XC94915. http://www.xeno-canto.org/94915.

Fisher, S. 2011. Eurasian blackcap (*Sylvia atricapilla*). XC77835. http://www.xeno-canto.org/77835.

Krabbe, N. 2011. Eurasian blackcap (*Sylvia atricapilla*). XC77466. http://www.xeno-canto.org/77466.

Grosselet, M. 2007. Eurasian blackcap (*Sylvia atricapilla*). XC55756. http://www.xeno-canto.org/55756.

Hansen, L.A. 2017. Eurasian blackcap (*Sylvia atricapilla*). XC378313. http://www.xeno-canto.org/378313.

Hussey, H. 2017. Eurasian blackcap (*Sylvia atricapilla*). XC364979. http://www.xeno-canto.org/364979.

M., D. Eurasian blackcap (*Sylvia atricapilla*). XC337261. http://www.xeno-canto.org/337261.

Szczypinski, P. 2014. Eurasian blackcap (*Sylvia atricapilla*). XC182600. http://www.xeno-canto.org/182600.

Grosselet, M. 2007. Eurasian blackcap (*Sylvia atricapilla*). XC55755. http://www.xeno-canto.org/55755.

Grosselet, M. 2017. Garden warbler (*Sylvia borin*). XC374739, XC374737. http://www.xeno-canto.org/374739.

Elman, U. 2016. Garden warbler (*Sylvia borin*). XC345786. http://www.xeno-canto.org/345786.

Grosselet, M. 2010. Garden warbler (*Sylvia borin*). XC54246. http://www.xeno-canto.org/54246.

Nefdt, R. 2012. Garden warbler (*Sylvia borin*). XC118446, XC118445, XC118444. http://www.xeno-canto.org/118446.

Elman, U. 2017. Common whitethroat (*Sylvia communis*). XC389008. http://www.xeno-canto.org/389008.

Litsgard, M. 2017. Common whitethroat (*Sylvia communis*). XC377005. http://www.xeno-canto.org/377005.

Litsgard, M. 2015. Common whitethroat (*Sylvia communis*). XC289803. http://www.xeno-canto.org/289803.

Elman, U. 2015. Common whitethroat (*Sylvia communis*). XC288934. http://www.xeno-canto.org/288934.

McGeehan, A. 2014. Common whitethroat (*Sylvia communis*). XC256109. http://www.xeno-canto.org/256109.

Mroczko, C. 2017. Common whitethroat (*Sylvia communis*). XC384995, XC384994. http://www.xeno-canto.org/384995.

Hussey, H. 2017. Common whitethroat (*Sylvia communis*). XC384002. http://www.xeno-canto.org/384002.

Hussey, H. 2015. Common whitethroat (*Sylvia communis*). XC290302. http://www.xeno-canto.org/290302.

Hansen, L.A. 2014. Common whitethroat (*Sylvia communis*). XC208277, XC208272. http://www.xeno-canto.org/208277.

Mroczko, C. 2017. Common whitethroat (*Sylvia communis*). XC384997, XC384996. http://www.xeno-canto.org/384997.

Lane, D. 2011. Rusty-collared seedeater (*Sporophila collaris*). XC75702. http://www.xeno-canto.org/75702.

Lopez-Lanus, B. Rusty-collared seedeater (*Sporophila collaris*). XC47158. http://www.xeno-canto.org/47158.

Gomez, O.H.M. 2009. Inca jay (*Cyanocorax yncas*). XC244732. http://www.xeno-canto.org/244732.

Cruickshank, I. 2014. Fox sparrow (*Passerella iliaca*). XC182418. http://www.xeno-canto.org/182418.

Matusiak, J. 2014. Brambling (*Fringilla montifringilla*). XC181546. http://www.xeno-canto.org/181546.

Lane, D. 2014. Baltimore oriole (*Icterus galbula*). XC175543, XC175542. http://www.xeno-canto.org/175543.

Calvet, J. 2014. Black-eared wheatear (*Oenanthe hispanica*). XC176144. http://www.xeno-canto.org/176144.

Peron, M. 2014. Black-eared wheatear (*Oenanthe hispanica*). XC178459. http://www.xeno-canto.org/178459.

Driver, P. 2012. Ruby-crowned kinglet (*Regulus calendula*). XC174939. http://www.xeno-canto.org/174939.

Amat, D.G. 2007. Austral thrush (*Turdus falcklandii magellanicus*). XC15293. http://www.xeno-canto.org/15293.

DaCosta, J. Dusky indigobird (*Vidua funerea*). XC41893, XC41892. http://www.xeno-canto.org/41893.

Bradley, J. and Bradley, D. 2012. Dusky indigobird (*Vidua funerea*). XC212710. http://www.xeno-canto.org/212710.

Lambert, F. 2008. Jambandu indigobird (*Vidua raricola*). XC24970. http://www.xeno-canto.org/24970.

DaCosta, J. 2009. Purple indigobird (*Vidua purpurascens*). XC41901, XC31860. http://www.xeno-canto.org/41901.

Peacock, F. 2015. Purple indigobird (*Vidua purpurascens*). XC307240. http://www.xeno-canto.org/307240.

Peacock, F. 2015. Village indigobird (*Vidua chalybeata*). XC307241. http://www.xeno-canto.org/307241.

Kilusu, I. 2017. Village indigobird (*Vidua chalybeata*). XC378617. http://www.xeno-canto.org/378617.

DaCosta, J. 2009. Village indigobird (*Vidua chalybeata*). XC41906. http://www.xeno-canto.org/41906.

DaCosta, J. 2008. Village indigobird (*Vidua chalybeata*). XC41904. http://www.xeno-canto.org/41904.

DaCosta, J. 2009. Zambezi indigobird (*Vidua codringtoni*). XC41897. http://www.xeno-canto.org/41897.

DaCosta, J. 2008. Zambezi indigobird (*Vidua codringtoni*). XC41896. http://www.xeno-canto.org/41896.

Lane, D. 2008. Grey seedeater (*Sporophila intermedia*). XC65978. http://www.xeno-canto.org/65978.

Spencer, A. 2009. Tooth-billed wren (*Odontorchilus cinereus*). XC38597. http://www.xeno-canto.org/38597.

Hoyer, R.C. 2006. Tooth-billed wren (*Odontorchilus cinereus*). XC17397. http://www.xeno-canto.org/17397.

Hansen, L.A. 1991. Abyssinian thrush (*Turdus abyssinicus*). XC124898. http://www.xeno-canto.org/124898.

Saucier, J.R. 2014. Yellow-legged thrush (*Turdus flavipes*). XC263396. http://www.xeno-canto.org/263396.

Spencer, A. 2011. Lawrence's thrush (*Turdus lawrencii*). XC122325. http://www.xeno-canto.org/122325.

Hennessey, A.B. 2001. Lawrence's thrush (*Turdus lawrencii*). XC3258. http://www.xeno-canto.org/3258.

Haribal, M. 2013. Lawrence's thrush (*Turdus lawrencii*). XC203473. http://www.xeno-canto.org/203473.

Haribal, M. 2010. Lawrence's thrush (*Turdus lawrencii*). XC154446. http://www.xeno-canto.org/154446.

Mark, T. 2012. Lawrence's thrush (*Turdus lawrencii*). XC118342. http://www.xeno-canto.org/118342.

Lane, D. 2015. Pale-breasted thrush (*Turdus leucomelas*). XC270254. http://www.xeno-canto.org/270254.

Bradley, J. 2011. African thrush (*Turdus pelios*). XC72879. http://www.xeno-canto.org/72879.

Lane, D. 2007. Reunion grey white-eye (*Zosterops borbonicus*). XC26322. http://www.xeno-canto.org/26322.

Nefdt, R. 2011. African yellow white-eye (*Zosterops senegalensis*). XC92067. http://www.xeno-canto.org/92067.

Nelson, K. 2015. Woodhouse's scrub jay (*Aphelocoma woodhouseii*). XC215457. http://www.xeno-canto.org/215457.

Macaulay Library:

Horne, J.F.M. 1974. Reunion grey white-eye. ML74648. https://macaulaylibrary.org/asset/74648.

Horne, J.F.M. 1973. Reunion grey white-eye. ML72208. https://macaulaylibrary.org/asset/72208.

Horne, J.F.M. 1994. African thrush. ML72170. https://macaulaylibrary.org/asset/72170.

Keller, G.A. 2001. Phainopepla. ML118622. https://macaulaylibrary.org/asset/118622.

Hershberger, W.L. 1999. Blue-grey gnatcatcher. ML100793, ML100792. https://macaulaylibrary.org/asset/100793.

Hershberger, W.L. 1999. Blackburnian warbler. ML100864, ML100863. https://macaulaylibrary.org/asset/100864.

Horne, J.F.M. 1982. Rüppell's robin-chat. ML66009. https://macaulaylibrary.org/asset/66009.

North, M.E.W. 1964. Rüppell's robin-chat. ML11257, ML11250. https://macaulaylibrary.org/asset/11257.

Marantz, C.A. 1998. Long-billed thrasher. ML112604, ML112603. https://macaulaylibrary.org/asset/112604.

Keller, G.A. 1995. Long-billed thrasher. ML105587, ML105582, ML105569, ML105550, ML105552. https://macaulaylibrary.org/asset/105587.

Medler, M.D. 1998. Long-billed thrasher. ML87424. https://macaulaylibrary.org/asset/87424.

Sada, A.M. 1993. Long-billed thrasher. ML86466. https://macaulaylibrary.org/asset/86466.

Marantz, C.A. 1993. Long-billed thrasher. ML76911. https://macaulaylibrary.org/asset/76911.

Budney, G.F.1995. Le Conte's thrasher. ML25286, ML25285, ML125284, ML125275, ML125274, ML125273, ML125263. https://macaulaylibrary.org/asset/125286.

Keller, G.A. 1999. Curve-billed thrasher. ML109077, ML109065. https://macaulaylibrary.org/asset/109077.

Allen, A.A. 1958. Curve-billed thrasher. ML11150. https://macaulaylibrary.org/asset/11150.

Budney, G.F. 1993. Sage thrasher. ML133149. https://macaulaylibrary.org/asset/133149.

Keller, G.A. 1993. Pine grosbeak. ML105297. https://macaulaylibrary.org/asset/105297.

Stein, R.C. 1961. Pine grosbeak. ML12995. https://macaulaylibrary.org/asset/12995.

Keller, G.A. 1989. Cassin's finch. ML4966, ML44965, ML44998, ML44973, ML44972, ML4971. https://macaulaylibrary.org/asset/44966.

Keller, G.A. 2002. Cassin's finch. ML120239, ML120238. https://macaulaylibrary.org/asset/120239.

Herr, D.S. 1993. Cassin's finch. ML107518. https://macaulaylibrary.org/asset/107518.

Little, R.S. 1994. Cassin's finch. ML106627. https://macaulaylibrary.org/asset/106627.

Hershberger, W.L. 1995. Cassin's finch. ML96373, ML73849, ML73825. https://macaulaylibrary.org/asset/96373.

Little, R.S. 1990. Cassin's finch. ML50750, ML50730. https://macaulaylibrary.org/asset/50750.

Keller, G.A. 1990. Cassin's finch. ML50295, ML50197. https://macaulaylibrary.org/asset/50295.

Little, R.S. 1989. Cassin's finch. ML45322. https://macaulaylibrary.org/asset/45322.

Herr, D.S. 1989. Cassin's finch. ML45296. https://macaulaylibrary.org/asset/45296.

Stein, R.C. 1961. Cassin's finch. ML12910. https://macaulaylibrary.org/asset/12910.

H. Douglas Pratt. 1978. Iiwi. ML5861, ML5859. https://macaulaylibrary.org/asset/5861.

H. Douglas Pratt. 1977. Iiwi. ML5288. https://macaulaylibrary.org/asset/5288.

Hershberger, W.L. 1999. Scarlet tanager. ML100801. https://macaulaylibrary.org/asset/100801.

Hershberger, W.L. 1999. White-eyed vireo. ML100814, ML100812. https://macaulaylibrary.org/asset/100814.

Geoffrey A. Keller. 1994. White-eyed vireo. ML105408. https://macaulaylibrary.org/asset/105408.

Horne, J.F.M. 1977. African yellow white-eye. ML79352. https://macaulaylibrary.org/asset/79352.

Horne, J.F.M. 1986. African yellow white-eye. ML55940. ttps://macaulaylibrary.org/asset/55940.

Horne, J.F.M. 1974. Mauritius grey white-eye. ML74634, ML74624, ML73391, ML73385, ML74629. https://macaulaylibrary.org/asset/74634.

Horne, J.F.M. 1974. Common myna. ML74545. https://macaulaylibrary.org/asset/74545.

North, M.E.W. 1964. Red-capped lark. ML8016. https://macaulaylibrary.org/asset/8016.

Kaestner, P.G. 1977. Snowy-crowned robin-chat. ML1111. https://macaulaylibrary.org/asset/1111.

Kaestner, P.G. 1978. Snowy-crowned robin-chat. ML1518. https://macaulaylibrary.org/asset/1518.

Hennessey, A.B. 1998. Epaulet oriole. ML87810. https://macaulaylibrary.org/asset/87810.

Moyer, D.C. 1994. Grey longbill. ML87333. https://macaulaylibrary.org/asset/87333.

Macaulay, L.R. 1993. Sabota lark. ML60999. https://macaulaylibrary.org/asset/60999.

Kaestner, P.G. 1978. Blue-shouldered robin-chat. ML1464. https://macaulaylibrary.org/asset/1464.

Freeman, B. 2012. Rusty mouse-warbler. ML182809, ML182806. https://macaulaylibrary.org/asset/182809.

Freeman, A.C. 2012. Rusty mouse-warbler. ML182723, 182679, 182640, 182611. https://macaulaylibrary.org/asset/182723.

Fulton, D. 2013. Rusty mouse-warbler. ML180116, 180110. https://macaulaylibrary.org/asset/180116.

Prate, T. 1975. Rusty mouse-warbler. ML169815. https://macaulaylibrary.org/asset/169815.

Freeman, B. 2011. Rusty mouse-warbler. ML166990, ML166989, ML166987, ML166908. https://macaulaylibrary.org/asset/166990.

Andrew, M. 1998. Rusty mouse-warbler. ML100478, ML100406, ML100346. https://macaulaylibrary.org/asset/100478.

Andrew, M. 1996. Rusty mouse-warbler. ML79874, ML79862. https://macaulaylibrary.org/asset/79874.

Connop, S. 1993. Rusty mouse-warbler. ML65837, ML65830. https://macaulaylibrary.org/asset/65837.

Horne, J.F.M. 1986. African yellow white-eye. ML55940. https://macaulaylibrary.org/asset/55940.

Hershberger, W.L. 1999. American robin. ML100920, ML100909. https://macaulaylibrary.org/asset/1999.

Sutton, R.L. 1989. Bahama mockingbird. ML164920. https://macaulaylibrary.org/asset/164920.

Barker, H. 1977. Black-headed grosbeak. ML25149. https://macaulaylibrary.org/asset/25149.

Hershberger, W.L. 1999. Blackburnian warbler. ML100864, ML100863. https://macaulaylibrary.org/asset/100864.

Little, R.S. and Kimball, J.W. 1962. Blue jay. ML13448. https://macaulaylibrary.org/asset/25149.

Little, R.S. 1987. Blue jay. ML49621. https://macaulaylibrary.org/asset/49621.

Elliott, L. 1987. Blue jay. ML38825. https://macaulaylibrary.org/asset/38825.

Allen, A.A. and Kellogg, P.P. 1952. Blue jay. ML13437. https://macaulaylibrary.org/asset/13437.

Crisologo, T.H. 2015. Blue jay. ML186883. https://macaulaylibrary.org/asset/186883.

Kellogg, P.P. and Stein, R.C. 1956. Blue jay. ML13443. https://macaulaylibrary.org/asset/13443.

Macaulay, L.R. 1991. Blue mockingbird. ML53722, ML53685. https://macaulaylibrary.org/asset/53722.

Hershberger, W.L. 1999. Blue-grey gnatcatcher. ML100793, ML100792. https://macaulaylibrary.org/asset/100793.

Kaestner, P.G. 1978. Blue-shouldered robin-chat. ML1464. https://macaulaylibrary.org/asset/1464.

Keller, G.A. 1989. Cassin's finch. ML44998. https://macaulaylibrary.org/asset/44998.

Keller, G.A. 2002. Cassin's finch. ML120292. https://macaulaylibrary.org/asset/120292.

Hite, J.M. 2013. Cassin's finch. ML181469. https://macaulaylibrary.org/asset/181469.

Macaulay, L.R. 1994. Chorister robin-chat. ML80635. https://macaulaylibrary.org/asset/80635.

Horne, J.F.M. 1974. Common myna. ML74545. https://macaulaylibrary.org/asset/74545.

Hennessey, A.B. 1998. Epaulet oriole. ML87810. https://macaulaylibrary.org/asset/87810.

Hershberger, W.L. 2000. Gray catbird. ML107405. https://macaulaylibrary.org/asset/107405.

McGowan, J.W. 2014. Gray catbird. ML192212. https://macaulaylibrary.org/asset/192212.

Medler, M.D. 1998. Gray catbird. ML93761. https://macaulaylibrary.org/asset/93761.

Medler, M.D. 2009. Gray catbird. ML140009. https://macaulaylibrary.org/asset/140009.

Macaulay, L.R. 1993. Large-billed lark. 69194. https://macaulaylibrary.org/asset/69194.

Robbins, M.B. 2016. Lesser gray shrike. ML524016. https://macaulaylibrary.org/asset/524016.

Horne, J.F.M. 1974. Mauritius white-eye. ML74624. https://macaulaylibrary.org/asset/74624.

Hershberger, W.L. 1999. Northern mockingbird. ML100752. https://macaulaylibrary.org/asset/100752.

Medler, M.D. 1998. Northern mockingbird. ML87477. https://macaulaylibrary.org/asset/87477.

Keller, G.A. 1999. Phainopepla. ML109065. https://macaulaylibrary.org/asset/109065.

McGowan, J.W. 2014. Purple finch. ML191062. https://macaulaylibrary.org/asset/191062.

Horne, J.F.M. 1982. Rüppell's robin-chat. ML66009. https://macaulaylibrary.org/asset/66009.

Horne, J.F.M. 1994. Rüppell's robin-chat. ML66003. https://macaulaylibrary.org/asset/66003.

North, M.E.W. 1964. Rüppell's robin-chat. ML11257. https://macaulaylibrary.org/asset/11257.

North, M.E.W. 1961. Rüppell's robin-chat. ML11250. https://macaulaylibrary.org/asset/11250.

Horne, J.F.M. 1974. Reunion gray white-eye. ML74648. https://macaulaylibrary.org/asset/74648.

Macaulay, L.R. 1993. Sabota lark. ML60999. https://macaulaylibrary.org/asset/60999.

Hershberger, W.L. 1999. Scarlet tanager. ML100801. https://macaulaylibrary.org/asset/100801.

Perkins, B. and Perkins, M. 1962. Steller's jay. ML13470. https://macaulaylibrary.org/asset/13470.

Serafin, L.A. 2002. Steller's jay. ML82353. https://macaulaylibrary.org/asset/82353.

Powys, V. 2008. Superb lyrebird. ML229696, ML229694, ML229675, ML229691, ML229674, ML229673, ML229697, ML229693, ML229666, ML229665, ML229663. https://macaulaylibrary.org/asset/229696.

Hobbs, Newton. Superb lyrebird. ML8003. https://macaulaylibrary.org/asset/8003.

Finch, D.W. 1990. Violaceous euphonia. ML57865. https://macaulaylibrary.org/asset/57865.

Hershberger, W.L. 1999. White-eyed vireo. ML100814, ML100812. https://macaulaylibrary.org/audio/100814.

Pratt, H.D. 1978. White-rumped shama. ML5829. http://macaulaylibrary.org/audio/5829.

Marantz, C.A. 1998. Long-billed thrasher. ML112604. http://macaulaylibrary.org/audio/112604.

Keller, G.A. 1995. Long-billed thrasher. ML105550. http://macaulaylibrary.org/audio/105550.

Sada, A.M. 1994. Long-billed thrasher. ML86491. http://macaulaylibrary.org/audio/86491.

McBride, C.S. 2000. Gray longbill. ML107828. https://macaulaylibrary.org/asset/107828.

Macaulay, L.R. 1999. Spiny babbler. ML108936. https://macaulaylibrary.org/asset/108936.

McChesney, M.P. 1956. Spotted palm thrush (Spotted morning-thrush). ML4259. http://macaulaylibrary.org/audio/4259.

Zimmerman, D.A. 1979. Spotted palm thrush (Spotted morning-thrush). ML21260. https://macaulaylibrary.org/asset/21260.

North, M.E.W. 1961. Snowy-crowned robin-chat. ML11241. https://macaulaylibrary.org/asset/11241.

North, M.E.W. 1962. Snowy-crowned robin-chat. ML11242, ML11239. https://macaulaylibrary.org/asset/11242.

Kaestner, P.G. 1978. Sooty chat. ML1504. https://macaulaylibrary.org/asset/1504.

Moyer, D.C. 1990. Orange ground thrush. ML101244. https://macaulaylibrary.org/asset/101244.

Medler, M.D. 2009. Black thrush. ML140003. https://macaulaylibrary.org/asset/140003.

Horne, J.F.M. 1994. African thrush. ML72170. Horne, J.F.M. 1974. Reunion grey white-eye. ML74648. https://macaulaylibrary.org/asset/74648.

Horne, J.F.M. 1973. Reunion grey white-eye. ML72208. https://macaulaylibrary.org/asset/72208.

Horne, J.F.M. 1994. African thrush. ML72170. https://macaulaylibrary.org/asset/72170.

Keller, G.A. 2001. Phainopepla. ML118622. https://macaulaylibrary.org/asset/118622.

Hershberger, W.L. 1999. Blue-grey gnatcatcher. ML100793, ML100792. https://macaulaylibrary.org/asset/100793.

Hershberger, W.L. 1999. Blackburnian warbler. ML100864, ML100863. https://macaulaylibrary.org/asset/100864.

Horne, J.F.M. 1982. Rüppell's robin-chat. ML66009. https://macaulaylibrary.org/asset/66009.

North, M.E.W. 1964. Rüppell's robin-chat. ML11257, ML11250. https://macaulaylibrary.org/asset/11257.

Marantz, C.A. 1998. Long-billed thrasher. ML112604, ML112603. https://macaulaylibrary.org/asset/112604.

Keller, G.A. 1995. Long-billed thrasher. ML105587, ML105582, ML105569, ML105550, ML105552. https://macaulaylibrary.org/asset/105587.

Medler, M.D. 1998. Long-billed thrasher. ML87424. https://macaulaylibrary.org/asset/87424.

Sada, A.M. 1993. Long-billed thrasher. ML86466. https://macaulaylibrary.org/asset/86466.

Marantz, C.A. 1993. Long-billed thrasher. ML76911. https://macaulaylibrary.org/asset/76911.

Budney, G.F.1995. Le Conte's thrasher. ML25286, ML25285, ML125284, ML125275, ML125274, ML125273, ML125263. https://macaulaylibrary.org/asset/125286.

Keller, G.A. 1999. Curve-billed thrasher. ML109077, ML109065. https://macaulaylibrary.org/asset/109077.

Allen, A.A. 1958. Curve-billed thrasher. ML11150. https://macaulaylibrary.org/asset/11150.

Budney, G.F. 1993. Sage thrasher. ML133149. https://macaulaylibrary.org/asset/133149.

Keller, G.A. 1993. Pine grosbeak. ML105297. https://macaulaylibrary.org/asset/105297.

Stein, R.C. 1961. Pine grosbeak. ML12995. https://macaulaylibrary.org/asset/12995.

Keller, G.A. 1989. Cassin's finch. ML4966, ML44965, ML44998, ML44973, ML44972, ML4971. https://macaulaylibrary.org/asset/44966.

Keller, G.A. 2002. Cassin's finch. ML120239, ML120238. https://macaulaylibrary.org/asset/120239.

Herr, D.S. 1993. Cassin's finch. ML107518. https://macaulaylibrary.org/asset/107518.

Little, R.S. 1994. Cassin's finch. ML106627. https://macaulaylibrary.org/asset/106627.

Hershberger, W.L. 1995. Cassin's finch. ML96373, ML73849, ML73825. https://macaulaylibrary.org/asset/96373.

Little, R.S. 1990. Cassin's finch. ML50750, ML50730. https://macaulaylibrary.org/asset/50750.

Keller, G.A. 1990. Cassin's finch. ML50295, ML50197. https://macaulaylibrary.org/asset/50295.

Little, R.S. 1989. Cassin's finch. ML45322. https://macaulaylibrary.org/asset/45322.

Herr, D.S. 1989. Cassin's finch. ML45296. https://macaulaylibrary.org/asset/45296.

Stein, R.C. 1961. Cassin's finch. ML12910. https://macaulaylibrary.org/asset/12910.

H. Douglas Pratt. 1978. Iiwi. ML5861, ML5859. https://macaulaylibrary.org/asset/5861.

H. Douglas Pratt. 1977. Iiwi. ML5288. https://macaulaylibrary.org/asset/5288.

Hershberger, W.L. 1999. Scarlet tanager. ML100801. https://macaulaylibrary.org/asset/100801.

Hershberger, W.L. 1999. White-eyed vireo. ML100814, ML100812. https://macaulaylibrary.org/asset/100814.

Geoffrey A. Keller. 1994. White-eyed vireo. ML105408. https://macaulaylibrary.org/asset/105408.

Horne, J.F.M. 1977. African yellow white-eye. ML79352. https://macaulaylibrary.org/asset/79352.

Horne, J.F.M. 1986. African yellow white-eye. ML55940. ttps://macaulaylibrary.org/asset/55940.

Horne, J.F.M. 1974. Mauritius grey white-eye. ML74634, ML74624, ML73391, ML73385, ML74629. https://macaulaylibrary.org/asset/74634.

Horne, J.F.M. 1974. Common myna. ML74545. https://macaulaylibrary.org/asset/74545.

North, M.E.W. 1964. Red-capped lark. ML8016. https://macaulaylibrary.org/asset/8016.

Kaestner, P.G. 1977. Snowy-crowned robin-chat. ML1111. https://macaulaylibrary.org/asset/1111.

Kaestner, P.G. 1978. Snowy-crowned robin-chat. ML1518. https://macaulaylibrary.org/asset/1518.

Hennessey, A.B. 1998. Epaulet oriole. ML87810. https://macaulaylibrary.org/asset/87810.

Moyer, D.C. 1994. Grey longbill. ML87333. https://macaulaylibrary.org/asset/87333.

Macaulay, L.R. 1993. Sabota lark. ML60999. https://macaulaylibrary.org/asset/60999.

Kaestner, P.G. 1978. Blue-shouldered robin-chat. ML1464. https://macaulaylibrary.org/asset/1464.

Freeman, B. 2012. Rusty mouse-warbler. ML182809, ML182806. https://macaulaylibrary.org/asset/182809.

Freeman, A.C. 2012. Rusty mouse-warbler. ML182723, 182679, 182640, 182611. https://macaulaylibrary.org/asset/182723.

Fulton, D. 2013. Rusty mouse-warbler. ML180116, 180110. https://macaulaylibrary.org/asset/180116.

Prate, T. 1975. Rusty mouse-warbler. ML169815. https://macaulaylibrary.org/asset/169815.

Freeman, B. 2011. Rusty mouse-warbler. ML166990, ML166989, ML166987, ML166908. https://macaulaylibrary.org/asset/166990.

Andrew, M. 1998. Rusty mouse-warbler. ML100478, ML100406, ML100346. https://macaulaylibrary.org/asset/100478.

Andrew, M. 1996. Rusty mouse-warbler. ML79874, ML79862. https://macaulaylibrary.org/asset/79874.

Connop, S. 1993. Rusty mouse-warbler. ML65837, ML65830. https://macaulaylibrary.org/asset/65837.

Horne, J.F.M. 1986. African yellow white-eye. ML55940. https://macaulaylibrary.org/asset/55940.

Hershberger, W.L. 1999. American robin. ML100920, ML100909. https://macaulaylibrary.org/asset/100920.

Sutton, R.L. 1989. Bahama mockingbird. ML164920. https://macaulaylibrary.org/asset/164920.

Barker, H. 1977. Black-headed grosbeak. ML25149. https://macaulaylibrary.org/asset/25149.

Hershberger, W.L. 1999. Blackburnian warbler. ML100864, ML100863. https://macaulaylibrary.org/asset/100864.

Little, R.S. and Kimball, J.W. 1962. Blue jay. ML13448. https://macaulaylibrary.org/asset/13448.

Little, R.S. 1987. Blue jay. ML49621. https://macaulaylibrary.org/asset/49621.

Elliott, L. 1987. Blue jay. ML38825. https://macaulaylibrary.org/asset/38825.

Allen, A.A. and Kellogg, P.P. 1952. Blue jay. ML13437. https://macaulaylibrary.org/asset/13437.

Crisologo, T.H. 2015. Blue jay. ML186883. https://macaulaylibrary.org/asset/186883.

Kellogg, P.P. and Stein, R.C. 1956. Blue jay. ML13443. https://macaulaylibrary.org/asset/13443.

Macaulay, L.R. 1991. Blue mockingbird. ML53722, ML53685. https://macaulaylibrary.org/asset/53722.

Hershberger, W.L. 1999. Blue-grey gnatcatcher. ML100793, ML100792. https://macaulaylibrary.org/asset/100793.

Kaestner, P.G. 1978. Blue-shouldered robin-chat. ML1464. https://macaulaylibrary.org/asset/1464.

Keller, G.A. 1989. Cassin's finch. ML44998. https://macaulaylibrary.org/asset/44998.

Keller, G.A. 2002. Cassin's finch. ML120292. https://macaulaylibrary.org/asset/120292.

Hite, J.M. 2013. Cassin's finch. ML181469. https://macaulaylibrary.org/asset/181469.

Macaulay, L.R. 1994. Chorister robin-chat. ML80635. https://macaulaylibrary.org/asset/80635.

Horne, J.F.M. 1974. Common myna. ML74545. https://macaulaylibrary.org/asset/74545.

Hennessey, A.B. 1998. Epaulet oriole. ML87810. https://macaulaylibrary.org/asset/87810.

Hershberger, W.L. 2000. Gray catbird. ML107405. https://macaulaylibrary.org/asset/107405.

McGowan, J.W. 2014. Gray catbird. ML192212. https://macaulaylibrary.org/asset/192212.

Medler, M.D. 1998. Gray catbird. ML93761. https://macaulaylibrary.org/asset/93761.

Medler, M.D. 2009. Gray catbird. ML140009. https://macaulaylibrary.org/asset/140009.

Macaulay, L.R. 1993. Large-billed lark. ML69194. https://macaulaylibrary.org/asset/69194.

Robbins, M.B. 2016. Lesser gray shrike. ML524016. https://macaulaylibrary.org/asset/524016.

Horne, J.F.M. 1974. Mauritius white-eye. ML74624. https://macaulaylibrary.org/asset/74624.

Hershberger, W.L. 1999. Northern mockingbird. ML100752. https://macaulaylibrary.org/asset/100752.

Medler, M.D. 1998. Northern mockingbird. ML87477. https://macaulaylibrary.org/asset/87477.

Keller, G.A. 1999. Phainopepla. ML109065. https://macaulaylibrary.org/asset/109065.

McGowan, J.W. 2014. Purple finch. ML191062. https://macaulaylibrary.org/asset/191062.

Horne, J.F.M. 1982. Rüppell's robin-chat. ML66009. https://macaulaylibrary.org/asset/66009.

Horne, J.F.M. 1994. Rüppell's robin-chat. ML66003. https://macaulaylibrary.org/asset/66003.

North, M.E.W. 1964. Rüppell's robin-chat. ML11257. https://macaulaylibrary.org/asset/11257.

North, M.E.W. 1961. Rüppell's robin-chat. ML11250. https://macaulaylibrary.org/asset/11250.

Horne, J.F.M. 1974. Reunion gray white-eye. ML74648. https://macaulaylibrary.org/asset/74648.

Macaulay, L.R. 1993. Sabota lark. ML60999. https://macaulaylibrary.org/asset/60999.

Hershberger, W.L. 1999. Scarlet tanager. ML100801. https://macaulaylibrary.org/asset/100801.

Perkins, B. and Perkins, M. 1962. Steller's jay. ML13470. https://macaulaylibrary.org/asset/13470.

Serafin, L.A. 2002. Steller's jay. ML82353. https://macaulaylibrary.org/asset/82353.

Powys, V. 2008. Superb lyrebird. ML229696, ML229694, ML229675, ML229691, ML229674, ML229673, ML229697, ML229693, ML229666, ML229665, ML229663. https://macaulaylibrary.org/asset/229696.

Hobbs, Newton. Superb lyrebird. ML8003. https://macaulaylibrary.org/asset/8003.

Finch, D.W. 1990. Violaceous euphonia. ML57865. https://macaulaylibrary.org/asset/57865.

Hershberger, W.L. 1999. White-eyed vireo. ML100814, ML100812. https://macaulaylibrary.org/audio/100814.

Pratt, H.D. 1978. White-rumped shama. ML5829. http://macaulaylibrary.org/audio/5829.

Marantz, C.A. 1998. Long-billed thrasher. ML112604. http://macaulaylibrary.org/audio/112604.

Keller, G.A. 1995. Long-billed thrasher. ML105550. http://macaulaylibrary.org/audio/105550.

Sada, A.M. 1994. Long-billed thrasher. ML86491. http://macaulaylibrary.org/audio/86491.

McBride, C.S. 2000. Gray longbill. ML107828. https://macaulaylibrary.org/asset/107828.

Macaulay, L.R. 1999. Spiny babbler. ML108936. https://macaulaylibrary.org/asset/108936.

McChesney, M.P. 1956. Spotted palm thrush (Spotted morning-thrush). ML4259. http://macaulaylibrary.org/audio/4259.

Zimmerman, D.A. 1979. Spotted palm thrush (Spotted morning-thrush). ML21260. https://macaulaylibrary.org/asset/21260.

North, M.E.W. 1961. Snowy-crowned robin-chat. ML11241. https://macaulaylibrary.org/asset/11241.

North, M.E.W. 1962. Snowy-crowned robin-chat. ML11242, ML11239. https://macaulaylibrary.org/asset/11242.

Kaestner, P.G. 1978. Sooty chat. ML1504. https://macaulaylibrary.org/asset/1504.

Moyer, D.C. 1990. Orange ground thrush. ML101244. https://macaulaylibrary.org/asset/101244.

Medler, M.D. 2009. Black thrush. ML140003. https://macaulaylibrary.org/asset/140003.

Horne, J.F.M. 1994. African thrush. ML72170. https://macaulaylibrary.org/asset/72170.
