# Supplementary figures and images for: Evolutionary origins of vocal mimicry in songbirds
Source: Evol Lett. 2018 Jun 22;2(4):417–26. doi: 10.1002/evl3.62 (PMC6121844; doi:10.1002/evl3.62)

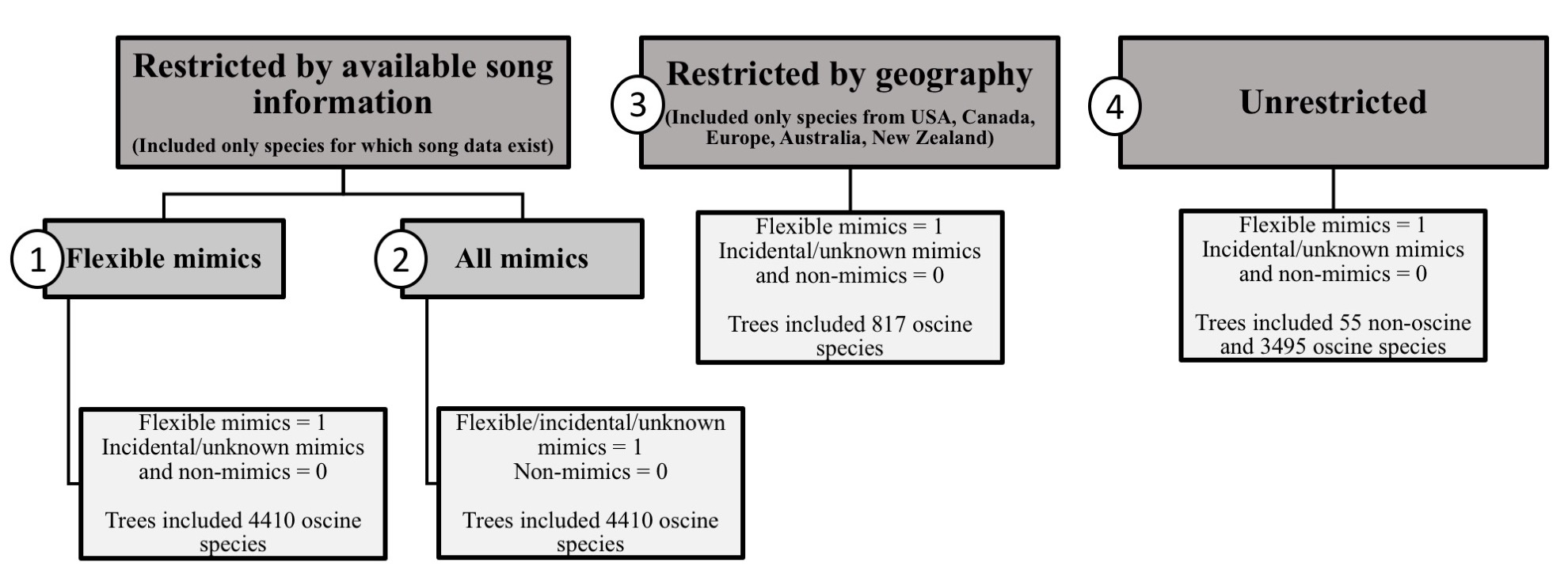

Supplement: Supplementary file 2 — Figure S1 [file EVL3-2-417-s002.tiff]
