# Supplementary material for: Evolutionary origins of vocal mimicry in songbirds
Source: Evol Lett. 2018 Jun 22;2(4):417–26. doi: 10.1002/evl3.62 (PMC6121844; doi:10.1002/evl3.62)
Supplement: Supplementary file 4 — Table S2 [file EVL3-2-417-s004.pdf]

Oscine family	Species (in analysis)	Species (total)	Proportion of species (in analysis)	Mimic species (flexible)	Proportion flexible mimic (of total)	Mimic species (all)	Proportion all mimic (of total)	Probability ancestor was mimic (flexible)	Probability ancestor was mimic (all)
Acanthizidae	59	65	0.91	12	0.18	18	0.28	0.008 ± 0.0	0.035 ± 0.0
Acrocephalidae	51	61	0.84	13	0.21	16	0.26	0	0
Alaudidae	85	97	0.88	32	0.33	35	0.36	0	0.002 ± 0.0
Artamidae	22	24	0.92	10	0.42	12	0.5	0.005 ± 0.0	0.077 ± 0.002
Atrichornithidae	2	2	1.00	1	0.5	2	1	**0.574 ± 0.005**	**0.92 ± 0.003**
Cardinalidae	53	70	0.76	2	0.03	2	0.03	0	0
Certhiidae	10	11	0.91	0	0	2	0.18	0	0.003 ± 0.0
Chloropseidae	8	11	0.73	4	0.36	4	0.36	0.046 ± 0.003	0.099 ± 0.003
Cisticolidae	145	158	0.92	1	0.01	2	0.01	0	0.001 ± 0.0
Corvidae	109	130	0.84	21	0.16	29	0.22	0	0
Dasyornithidae	3	3	1.00	0	0	1	0.33	0	0.096 ± 0.002
Dicaeidae	43	48	0.90	1	0.02	1	0.02	0	0
Dicruridae	21	25	0.84	13	0.52	17	0.68	**0.854 ± 0.006**	**0.831 ± 0.005**
Emberizidae	157	180	0.87	4	0.02	16	0.09	0	0
Estrildidae	130	141	0.92	0	0	1	0.01	0	0
Fringillidae	184	225	0.82	23	0.10	42	0.19	0	0.003 ± 0.0
Icteridae	100	109	0.92	7	0.06	20	0.18	0	0
Laniidae	29	33	0.88	12	0.36	13	0.39	0	0.002 ± 0.0
Leiothrichidae	121	133	0.91	4	0.03	7	0.05	0	0
Locustellidae	53	62	0.85	0	0	2	0.03	0	0
Macrosphenidae	17	18	0.94	1	0.06	2	0.11	0	0.001 ± 0.0
Malaconotidae	46	49	0.94	1	0.02	2	0.04	0	0
Meliphagidae	164	187	0.88	3	0.02	13	0.07	0	0
Menuridae	2	2	1.00	2	1	2	1	**0.949 ± 0.002**	**0.926 ± 0.003**
Mimidae	34	34	1.00	16	0.47	17	0.5	**0.742 ± 0.004**	**0.635 ± 0.003**
Monarchidae	82	101	0.81	0	0	1	0.01	0	0
Motacillidae	61	66	0.92	3	0.05	8	0.12	0	0.001 ± 0.0
Muscicapidae	273	321	0.85	54	0.17	82	0.26	0	0
Nectariniidae	124	143	0.87	6	0.04	9	0.06	0	0
Nicatoridae	3	3	1.00	2	0.67	2	0.67	**0.417 ± 0.007**	**0.480 ± 0.006**
Oriolidae	29	38	0.76	5	0.13	6	0.16	0	0.001 ± 0.0
Orthonychidae	3	3	1.00	0	0	1	0.33	0	0.107 ± 0.001
Pachycephalidae	41	56	0.73	3	0.05	4	0.07	0	0.001 ± 0.0
Paradisaeidae	27	41	0.66	0	0	2	0.05	0	0.002 ± 0.0
Paridae	53	64	0.83	1	0.02	2	0.03	0	0.004 ± 0.0
Parulidae	104	119	0.87	5	0.04	10	0.08	0.001 ± 0.0	0.002 ± 0.0
Passeridae	36	51	0.71	0	0	2	0.04	0	0.002 ± 0.0
Phylloscopidae	71	77	0.92	0	0	3	0.04	0	0
Platysteiridae	29	33	0.88	2	0.06	2	0.06	0	0.002 ± 0.0
Polioptilidae	15	18	0.83	3	0.17	4	0.22	0	0.003 ± 0.0
Ptiliogonatidae	4	4	1.00	1	0.25	2	0.5	0.002 ± 0.0	0.088 ± 0.001
Ptilonorhynchidae	17	20	0.85	14	0.7	17	0.85	**0.576 ± 0.008**	**0.748 ± 0.005**
Pycnonotidae	117	151	0.77	2	0.01	5	0.03	0	0
Regulidae	6	6	1.00	0	0	3	0.5	0	0.091 ± 0.001
Rhipiduridae	42	50	0.84	1	0.02	1	0.02	0	0.001 ± 0.0
Stenostiridae	9	9	1.00	1	0.11	4	0.44	0	0.009 ± 0.0
Sturnidae	108	123	0.88	14	0.11	22	0.18	0	0.002 ± 0.0
Sylviidae	56	70	0.80	6	0.09	11	0.16	0	0
Thraupidae	373	374	1.00	2	0.01	9	0.02	0	0
Troglodytidae	76	84	0.90	2	0.02	7	0.08	0	0
Turdidae	141	167	0.84	19	0.11	29	0.17	0	0
Vangidae	21	21	1.00	0	0	1	0.05	0	0
Viduidae	20	20	1.00	0	0	17	0.85	0	0.081 ± 0.001
Vireonidae	58	63	0.92	2	0.03	5	0.08	0	0
Zosteropidae	100	127	0.79	7	0.06	7	0.06	0	0
**Totals**	**3799**	**5045**	**Avg = 0.75**	**338**	**Avg = 0.07**	**556**	**Avg = 0.11**	n/a	n/a
