# Supplementary material for: Evolutionary origins of vocal mimicry in songbirds
Source: Evol Lett. 2018 Jun 22;2(4):417–26. doi: 10.1002/evl3.62 (PMC6121844; doi:10.1002/evl3.62)
Supplement: Supplementary file 5 — Supporting information [file EVL3-2-417-s005.docx]

**Supplemental Methods**

***Regional analysis***

Species included in the regional analysis were limited to oscines from North America, Europe, and Australia. We assumed the song of all oscine species from these regions has been described, as the avifauna in these areas has been relatively well-studied. As such, we did not expect any undocumented mimic species and were confident in our characterization of presence or absence of mimicry. Only species classified as flexible mimics were scored as mimics in analysis. All other species (incidental and unknown mimics, non-mimics) were scored as mimicry absent.

Binary vocal imitation scores were mapped onto phylogenetic trees taken from the global phylogeny of birds accompanying Jetz et al. (2012). We downloaded 1,000 trees that included 817 oscine species, 130 of which were mimics. We ran the same ancestral state reconstruction as used on the flexible and all-mimics datasets.

***Global analysis***

In this analysis, we pruned the Hackett All Species set of trees (Jetz et al 2012) to include a variety of nonoscine and oscine species. First, we removed all but one species from each order other than Passeriformes. These non-oscine species were included solely as outgroups. Second, we removed all but one species from each suboscine family within Passeriformes as suboscines are not known to be capable of vocal learning. Third, we excised all but one species from each genus within oscine families with no flexible mimics. We retained all species from oscine families that included at least one mimetic species. The final pruned trees contained 41 nonpasseriform species, 14 suboscine species, and 3,495 oscine species. Only species classified as flexible mimics were scored as mimics in analysis. All other species (incidental and unknown mimics, non-mimics) were scored as mimicry absent.

Binary vocal imitation scores were mapped onto phylogenetic trees taken from the global phylogeny of birds accompanying Jetz et al. (2012). We ran the same ancestral state reconstruction as used on the flexible and all mimics datasets.
